# The pyramid representation of the functional network using resting-state fMRI

**DOI:** 10.1093/psyrad/kkac011

**Published:** 2022-11-12

**Authors:** Zhipeng Yang, Luying Li, Yaxi Peng, Yuanyuan Qin, Muwei Li

**Affiliations:** College of Electronic Engineering, Chengdu University of Information Technology, Chengdu, Sichuan 610225, China; College of Electronic Engineering, Chengdu University of Information Technology, Chengdu, Sichuan 610225, China; College of Electronic Engineering, Chengdu University of Information Technology, Chengdu, Sichuan 610225, China; Department of Radiology, Tongji Hospital, Tongji Medical College, Huazhong University of Science and Technology, Wuhan, Hubei 430000, China; Vanderbilt University Institute of Imaging Science, Vanderbilt University, Nashville, TN 37232, USA

**Keywords:** multi-scale, functional network, graph theory, resting-state fMRI, support vector machine, Alzheimer's disease

## Abstract

**Background:**

Resting-state functional magnetic resonance imaging (RS-fMRI) has been proved to be a useful tool to study the brain mechanism in the quest to probe the distinct pattern of inter-region interactions in the brain. As an important application of RS-fMRI, the graph-based approach characterizes the brain as a complex network. However, the network is susceptible to its scale that determines the trade-off between sensitivity and anatomical variability.

**Objective:**

To balance sensitivity and anatomical variability, a pyramid representation of the functional network is proposed, which is composed of five individual networks reconstructed at multiple scales.

**Methods:**

The pyramid representation of the functional network was applied to two groups of participants, including patients with Alzheimer's disease (AD) and normal elderly (NC) individuals, as a demonstration. Features were extracted from the multi-scale networks and were evaluated with their inter-group differences between AD and NC, as well as the discriminative power in recognizing AD. Moreover, the proposed method was also validated by another dataset from people with autism.

**Results:**

The different features reflect the highest sensitivity to distinguish AD at different scales. In addition, the combined features have higher accuracy than any single scale-based feature. These findings highlight the potential use of multi-scale features as markers of the disrupted topological organization in AD networks.

**Conclusion:**

We believe that multi-scale metrics could provide a more comprehensive characterization of the functional network and thus provide a promising solution for representing the underlying functional mechanism in the human brain on a multi-scale basis.

## Introduction

Magnetic resonance imaging (MRI) techniques have been recognized as a valuable tool for improving our understanding of the pathological nature of human brain. Recently, resting-state functional MRI (RS-fMRI) has drawn considerable attention due to its capability for measuring spontaneous fluctuations in blood oxygen level-dependent (BOLD) signals (Biswal *et al*., 1995). A graph theory-based approach, as an important application of RS-fMRI, models the brain as a functional network constituted by nodes and edges (Wang *et al*., 2010). A node is often defined as a region of interest (ROI), grouping voxels that share similar anatomical characteristics. An edge, which is typically referred to as functional connectivity, represents the temporal relationship between the pairs of nodes (He *et al*., 2008; Supekar *et al*., 2008). The functional abnormalities observed in patients could thus be implicit changes in connectivity patterns (Dai *et al*., 2015; Delbeuck *et al*., 2003), which are frequently described by several essential metrics derived from the graph, such as the clustering coefficient (Holland & Leinhardt, 2015), efficiency (Latora & Marchiori, 2001), and node betweenness centrality (Barrat *et al*., 2004). These metrics could presumably provide conceivable biological markers that will improve our understanding of the functional mechanism of various neurological or psychiatric diseases. However, for the same individual, these metrics are altered during network remodeling based on different definitions of nodes (Wang *et al*., 2009). According to previous literature, a node is either defined as an anatomical structure or a single voxel. Meanwhile, the number of the nodes has been reported to range from 10 to 20 000 or even higher (at 4 mm resolution) (Fair *et al*., 2008; Valencia *et al*., 2009). Therefore, a consensus regarding the node definition is still lacking, which leads to the variations in the scale, size, complexity, and consequently the connectivity patterns specific to normal or abnormal functional networks.

Node definitions are frequently obtained by first registering the image from its native space to standard coordinates [e.g. the Montreal Neurological Institute space (MNI) (Evans *et al*., 1991)], and then superimposing a predefined atlas to parcellate the image. The sizes of the nodes, which determine the scale of the network, rely on the granularity of the atlas. The network thus could be characterized as, for example, a 90 × 90 matrix according to Automated Anatomical Labeling (AAL) atlas (Tzourio-Mazoyer *et al*., 2002), whose gray matter was presegmented to 90 structures, compared with a 70 000 × 70 000 matrix in line with voxel-level nodes (at 3 mm resolution in MNI). The large-scale network enables a more straightforward sorting of the nodes, such that the *ith* node specifies the same anatomical structure across the population (Djamanakova *et al*., 2014) and consequently increases the statistical 3 power, as brains share the similar characteristic at the same location. However, the large-scale network is also considered biased because the average measurement within a large anatomical structure is not sensitive to localized information. In contrast, the small-scale nodes are more effective because they are capable of capturing abnormalities in small focal areas. However, their limitations are also obvious. For one thing, the time courses measured in small-size nodes display a reduced signal-to-noise ratio (SNR). The segmentation of a brain into smaller parcels implies a increasing number of nodes, for example, 70 000 nodes over the brain in extreme cases. Therefore, the issue of noise is amplified because the network includes 70 000 noisy observations. Moreover, the boundary definitions of smaller nodes are less accurate, generally due to the registration error caused by the changes in cortical morphology, as well as the local heterogeneity in specific brain structures.

In this study, we propose multi-scale networks based on a set of multi-granularity brain atlases (Djamanakova *et al*., 2014). The images could be simultaneously segmented to yield 4, 6, 16, 58 and 84 gray matter nodes. Graph-based networks could then be reconstructed by measuring the temporal correlations between every pair of nodes at five scales. This approach enables the construction of a pyramid representation of the functional networks in the brain, in which the smallest scale graph is located at the bottom of the pyramid and the larger scale graphs are stacked one on top of the other. Unlike the pyramid model in digital imaging processing that consists of a series of copies of the original image with decreased resolution (Adelson *et al*., 1984), the proposed approach generates “down-sampled” graphs using a multi-granularity node definition. We applied the approach to two groups of participants, including patients with Alzheimer's disease (AD) and normal elderly (NC) individuals, to evaluate the discriminative ability of the multi-scale metrics, which are considered the features that could characterize the disease-specific disruption of the networks, in recognizing AD. We hypothesized that the pyramid representation would provide a more comprehensive characterization of the functional network because it encodes the most sensitive feature at every scale and could provide a better trade-off between feature sensitivity and anatomical variability.

## Methods

### Statement of ethics

The present study was approved by the Institutional Review Board of Tongji Hospital. Informed consent documents were obtained from the participants or their family members.

### Participants and image acquisition

Two groups of participants were recruited: (i) 21 patients who were diagnosed with probable AD using the NINCDS-ADRDA Alzheimer's Criteria (McKhann *et al*., 1984) and (ii) 20 healthy elderly participants with no history of psychiatric or neurological disorders. The demographic characteristics, including age, sex, and Mini-Mental State Examination (MMSE) of the recruited participants, are shown in Table 1.

**Table 1:** The demographics and characteristics of the selected population.

Group	Number	Sex	Age	MMSE
AD	21	10M/11F	66.8 ± 10.4	17.5 ± 3.6
NC	20	10M/10F	65.3 ± 7.3	28.5 ± 0.7
Differences	…	*P* = 0.88	*P* = 0.29	*P* < 0.0001

The inter-group differences in age and MMSE were assessed with Student's *t*-test. The differences in sex were evaluated by a Pearson Chi-square test.

A 3 Tesla MR scanner (GE Healthcare, USA) with an eight-channel head array coil was used in the present study. Each participant was scanned in a supine, head-first position. To ensure stability, cushions were placed symmetrically on both sides of the head. The participants were instructed to close their eyes, think of nothing in particular, and stay awake during the RS-fMRI scan. A gradient-echo planar sequence was performed to acquire the RS-fMRI images using the following parameters: repetition time (TR) = 2000 ms, echo time (TE) = 30 ms, matrix = 64 × 64, slices = 33, slice thickness = 4.0 mm with 0.5 mm gap, field of view (FOV) = 240 × 240 mm, and scan time = 8 min. A three-dimensional fast spoiled gradient-recalled-echo sequence was used to acquire structural data with a TR = 6.5 ms, TE = 2.1 ms, matrix = 256 × 256, slice thickness = 1.0 mm (0 gap), FOV = 256 × 256 mm, and scan time = 4 min 8 s.

In the Autism experiment, the data collected by the University of Pittsburgh School of Medicine on the Autism Brain Imaging Data Exchange website were used. Thirty patients diagnosed with autism, according to the Automation Diagnostic Observation Schedule-General (Lord *et al*., 2000) and expert clinical opinion, aged between 7 and 35 years, and 29 healthy individuals with no history of head trauma, birth complications, seizures, or psychiatric disorder, were used as control groups for the experiment.

During the data acquisition, participants were asked to chose their eyes and not to fall asleep, and a rapid gradient-echo planar sequence was performed to acquire the fMRI images using the following parameters: repetition time (TR) = 1 500 ms, echo time (TE) = 25 ms, slices = 29, slice thickness = 4.0 mm with a 0.5 mm gap, field of view (FOV) = 200  × 200 mm, and scan time = 5 min 9 s.

### Preprocessing

BOLD images were preprocessed using the statistical parametric mapping toolbox (SPM12: www.fil.ion.ucl.ac.uk/spm/software). First, the fMRI images were corrected for the intra-scan acquisition time differences and inter-scan head motions. Then, the corrected images were spatially smoothed with a Gaussian kernel (4 × 4 × 4 mm^3^ full-width at half-maximum). The time courses were corrected for signal drift and subsequently filtered with a bandpass filter (0.01–0.1 Hz) to reduce the high-frequency nonneuronal noise. The head motion artifacts were removed by performing multiple regressions of 24 motion parameters (Friston *et al*., 1996). Then, the mean CSF as well as white matter signals were regressed out to eliminate the physiological noise using a toolbox called REST (Song *et al*., 2011). Finally, the resulting images were registered to the MNI space to match the coordinates of the atlas.

### Multi-granularity atlas

**Figure 1:**
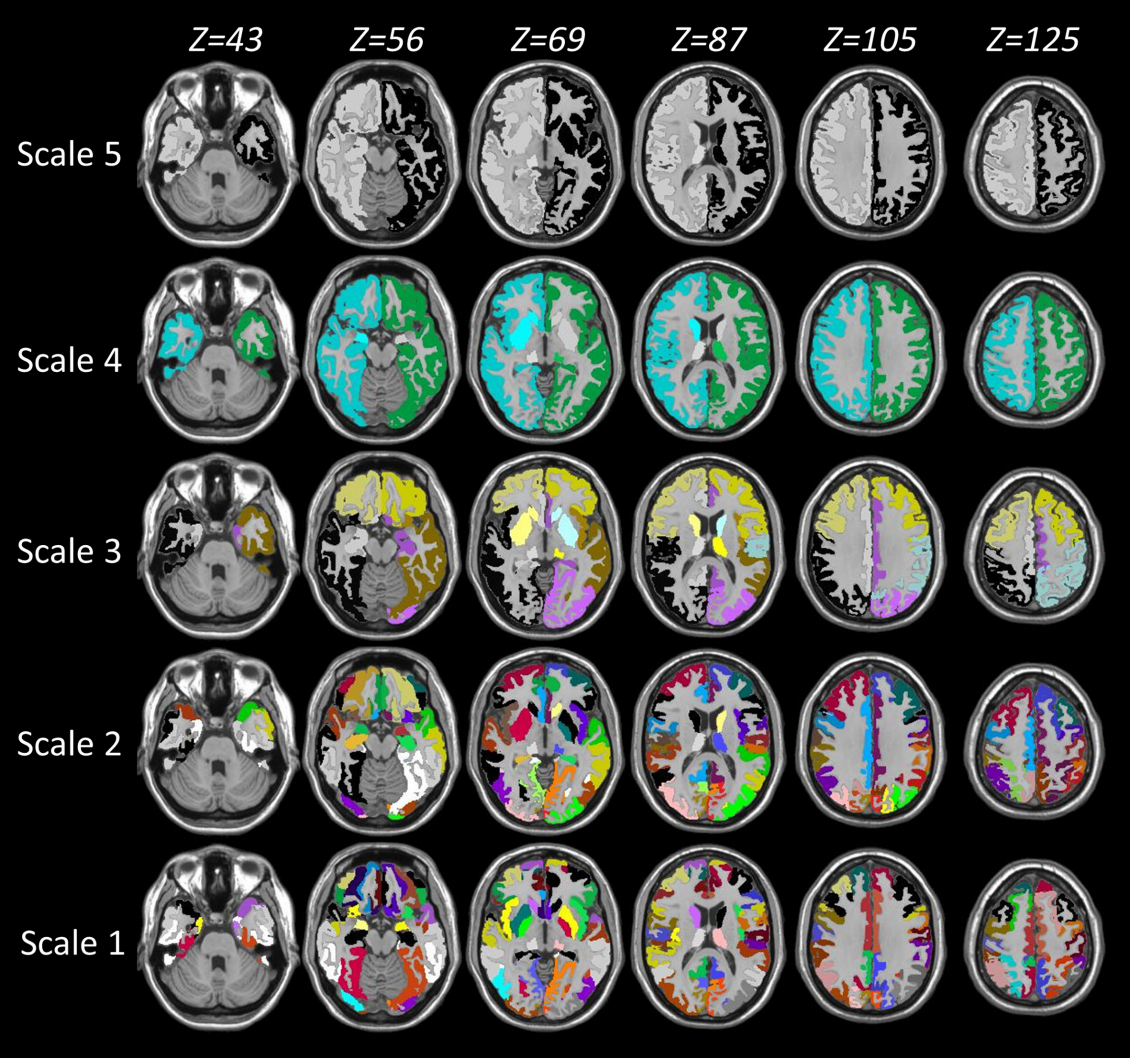
Segmentation results at five scales. The gray matter was segmented to 84, 58, 16, 6, and 4 parcels from Scale-1 to Scale-5 with each parcel displayed in a specific color.

The details of the multi-granularity atlas are presented in another study (Djamanakova *et al*., 2014). Briefly, 286 parcels that represent the highest granularity (Level-5) were manually defined in the MNI space. According to their ontological relationship, these parcels were then recombined hierarchically to reconstruct the Level-4, Level-3, Level-2, and Level-1 atlases, which separately consisted of 134, 54, 11, and 6 parcels over the entire brain. The Level-5 atlas corresponds to the smallest scale (Scale-1), while the Level-1 atlas corresponds to largest scale. “Scale” is used as the unit of the anatomical resolution throughout the paper to unify the description. As shown in Fig. 1, a structural image that was spatially normalized to the MNI space was segmented into multiple anatomical structures at five scales. In this study, since only gray matter areas were used to establish the functional network, the parcels, which are known as nodes in a graph, were further reduced to 84, 58, 16, 6, and 4. The lookup table of the node names is presented in Table 2.

**Table 2:** Names of the anatomical nodes at five scales.

Scale-5		Scale-2				Scale-1			
1	Telencephalon_L	1	SFG_L	43	Cingulate_L	1	SFG_L	43	ITG_L
2	Telencephalon_R	2	SFG_R	44	Cingulate_R	2	SFG_R	44	ITG_R
3	Diencephalon_L	3	MFG_L	45	Insula_L	3	SFG_PFC_L	45	PHG_L
4	Diencephalon_L	4	MFG_R	46	Insula_R	4	SFG_PFC_R	46	PHG_R
		5	IFG_L	47	Amyg_L	5	SFG_pole_L	47	ENT_L
		6	IFG_R	48	Amyg_R	6	SFG_pole_R	48	ENT_R
		7	OG_L	49	Hippo_L	7	MFG_L	49	FuG_L
		8	OG_R	50	Hippo_R	8	MFG_R	50	FuG_R
		9	RG_L	51	Caud_L	9	MFG_DPFC_L	51	SOG_L
		10	RG_R	52	Caud_R	10	MFG_DPFC_R	52	SOG_R
Scale-4		11	PoCG_L	53	Put_L	11	IFG_opercularis_L	53	MOG_L
1	CerebralCortex_L	12	PoCG_R	54	Put_R	12	IFG_opercularis_R	54	MOG_R
2	CerebralCortex_R	13	PrCG_L	55	GP_L	13	IFG_orbitalis_L	55	IOG_L
3	CerebralNucli_L	14	PrCG_R	56	GP_R	14	IFG_orbitalis_R	56	IOG_R
4	CerebralNucli_R	15	SPG_L	57	Thalamus_L	15	IFG_triangularis_L	57	Cu_L
5	Thalamus_L	16	SPG_R	58	Thalamus_R	16	IFG_triangularis_R	58	Cu_R
6	Thalamus_R	17	SMG_L			17	LFOG_L	59	LG_L
		18	SMG_R			18	LFOG_R	60	LG_R
		19	AG_L			19	MFOG_L	61	rostral_L
		20	AG_R			20	MFOG_R	62	rostral_R
		21	PrCu_L			21	RG_L	63	subcallosal_L
		22	PrCu_R			22	RG_R	64	subcallosal_R
		23	STG_L			23	PoCG_L	65	subgenual_ACC_L
		24	STG_R			24	PoCG_R	66	subgenual_ACC_R
		25	MTG_L			25	PrCG_L	67	dorsal_ACC_L
Scale-3		26	MTG_R			26	PrCG_R	68	dorsal_ACC_R
1	Frontal_L	27	ITG_L			27	SPG_L	69	PCC_L
2	Frontal_R	28	ITG_R			28	SPG_R	70	PCC_R
3	Parietal_L	29	Limbic_L			29	SMG_L	71	Insula_L
4	Parietal_R	30	Limbic_R			30	SMG_R	72	Insula_R
5	Temporal_L	31	FuG_L			31	AG_L	73	Amyg_L
6	Temporal_R	32	FuG_R			32	AG_R	74	Amyg_R
7	Limbic_L	33	SOG_L			33	PrCu_L	75	Hippo_L
8	Limbic_R	34	SOG_R			34	PrCu_R	76	Hippo_R
9	Occipital_L	35	MOG_L			35	STG_L	77	Caud_L
10	Occipital_R	36	MOG_R			36	STG_R	78	Caud_R
11	Insula_L	37	IOG_L			37	STG_L_pole	79	Put_L
12	Insula_R	38	IOG_R			38	STG_R_pole	80	Put_R
13	BasslGang_L	39	Cu_L			39	MTG_L	81	GP_L
14	BasslGang_R	40	Cu_R			40	MTG_R	82	GP_R
15	Thalamus_L	41	LG_L			41	MTG_L_pole	83	Thalamus_L
16	Thalamus_R	42	LG_R			42	MTG_R_pole	84	Thalamus_R

### Multi-scale graph-based functional networks

In each participant, average BOLD time courses were extracted from nodes. The functional connectivity *D_ij_* between each pair of nodes (*i* and *j*) was calculated using Pearson's correlation coefficients. Therefore, for each participant, we obtained five-scale functional networks separately characterized by 84 × 84, 58 × 58, 16 × 16, 6 × 6, and 4 × 4 symmetric matrices, with the value of the element (*i*, *j*) equivalent to *D_ij_*. The matrices were binarized by preserving a proportion *P* = 50% of the strongest connectivity. The rest elements as well as the main-diagonal elements (self-self connections) were set to 0.

Based on graph theory, several metrics, including the clustering coefficient, node betweenness centrality, and network efficiency, were calculated from the functional networks using a MATLAB-based package, namely, the brain connectivity toolbox (Rubinov & Sporns, 2010). Specifically, the clustering coefficient *C*_*i*_ of node *i* measures the interconnectivity among a group of neighboring nodes of *i*. The global clustering coefficient *C* is the mean of all *C*_*i*_ values. The global efficiency *E* is the mean of the inverse shortest path lengths of the network. The local efficiency *E_i_* is the *E* computed in the neighborhood of node *i*, which is considered a measure of how efficiently node *i* exchanges information. The node betweenness centrality *B_i_* is the fraction of shortest paths where a given node *i* is involved. Note that a definition of global betweenness centrality is not available.

### Validation of the multi-scale metrics

The multi-scale metrics were expanded to form 84-, 58-, 16-, 6-, and 4-dimensional feature vectors to characterize the functional architecture of each participant. At each scale, the feature vectors of all training samples, along with their known clinical labels (AD = 1, NC = −1), were used to train a classifier, namely a support vector machine (SVM) (Chang & Lin, 2011). Then a group of testing samples, whose clinical labels were “unknown,” could be mapped to the high-dimensional feature space and subsequently assigned to the appropriate categories by the trained classifier. The classification performance was evaluated by 10-fold cross-validation, which divided all samples into 10 subgroups and iteratively selected one subgroup as the testing group that did not participate in the training process. The specificity, sensitivity, and the accuracy of the proposed features could be obtained based on the classification results regarding each of the 10 subgroups that were tested. The feature dimension was further extended to 168 after concatenating all scales of features for each participant. The combined features were then sent to SVM for training and testing using the same validation procedure.

The amplitude of low-frequency fluctuations (ALFF) map (Zang *et al*., 2007) was calculated on a voxel basis from each fMRI image by summing the total amplitudes within the [0.01–0.1 Hz] frequency band. Then, the ALFF was normalized to *z*-score across the whole brain. For each participant, the five-scale features and the combined feature (of five scales) were also extracted from the ALFF map by averaging the ALFF value within each node. Then, the discriminative capability of the ALFF-based features was examined using the same cross-validation procedures.

## Results

### Multi-scale graph-based functional networks

**Figure 2:**
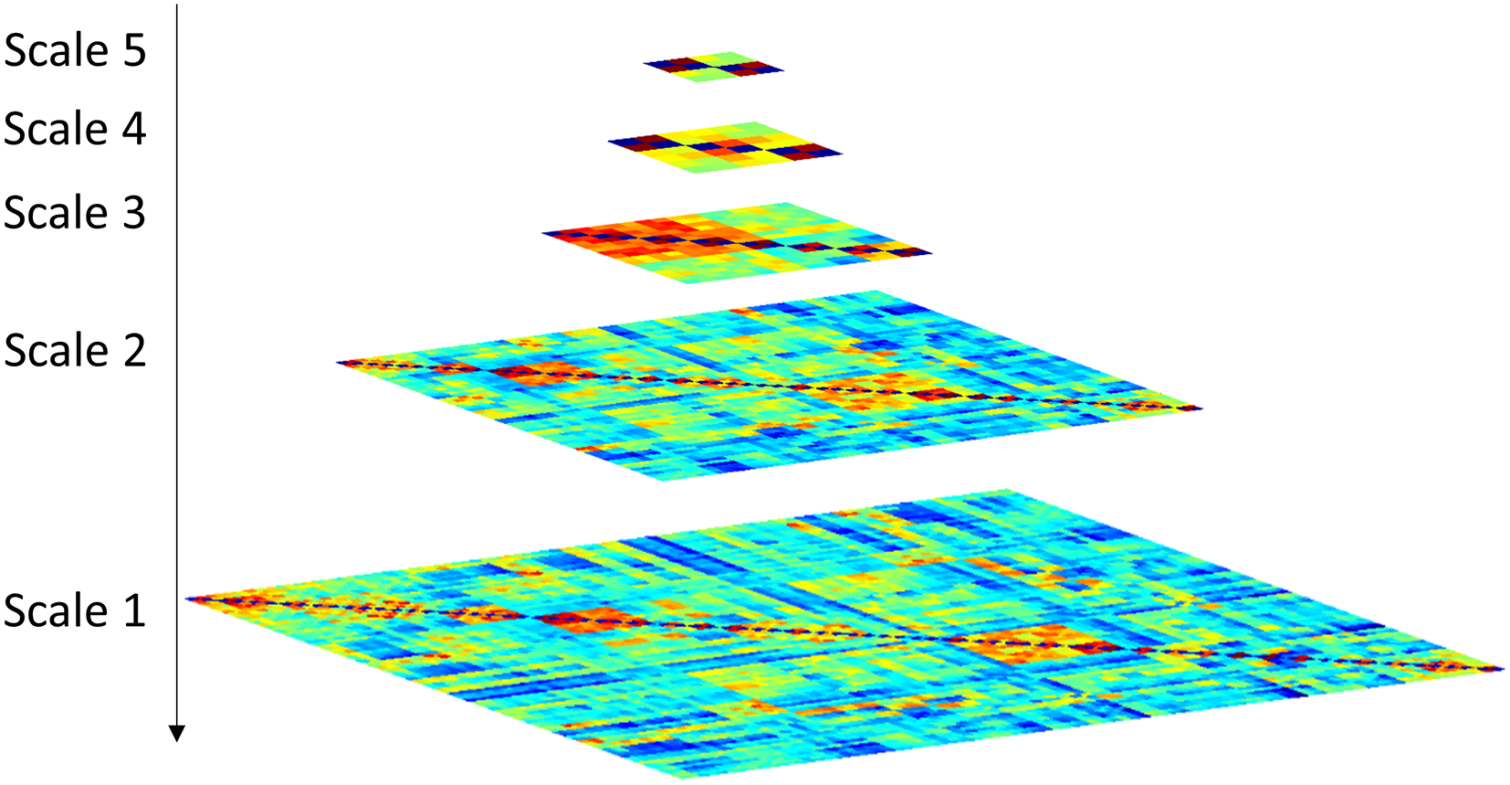
Pyramid representation of the multi-scale functional networks, with the smallest-scale graph on the bottom and the increased-scale of graphs stacked one on top of the other.

**Figure 3:**
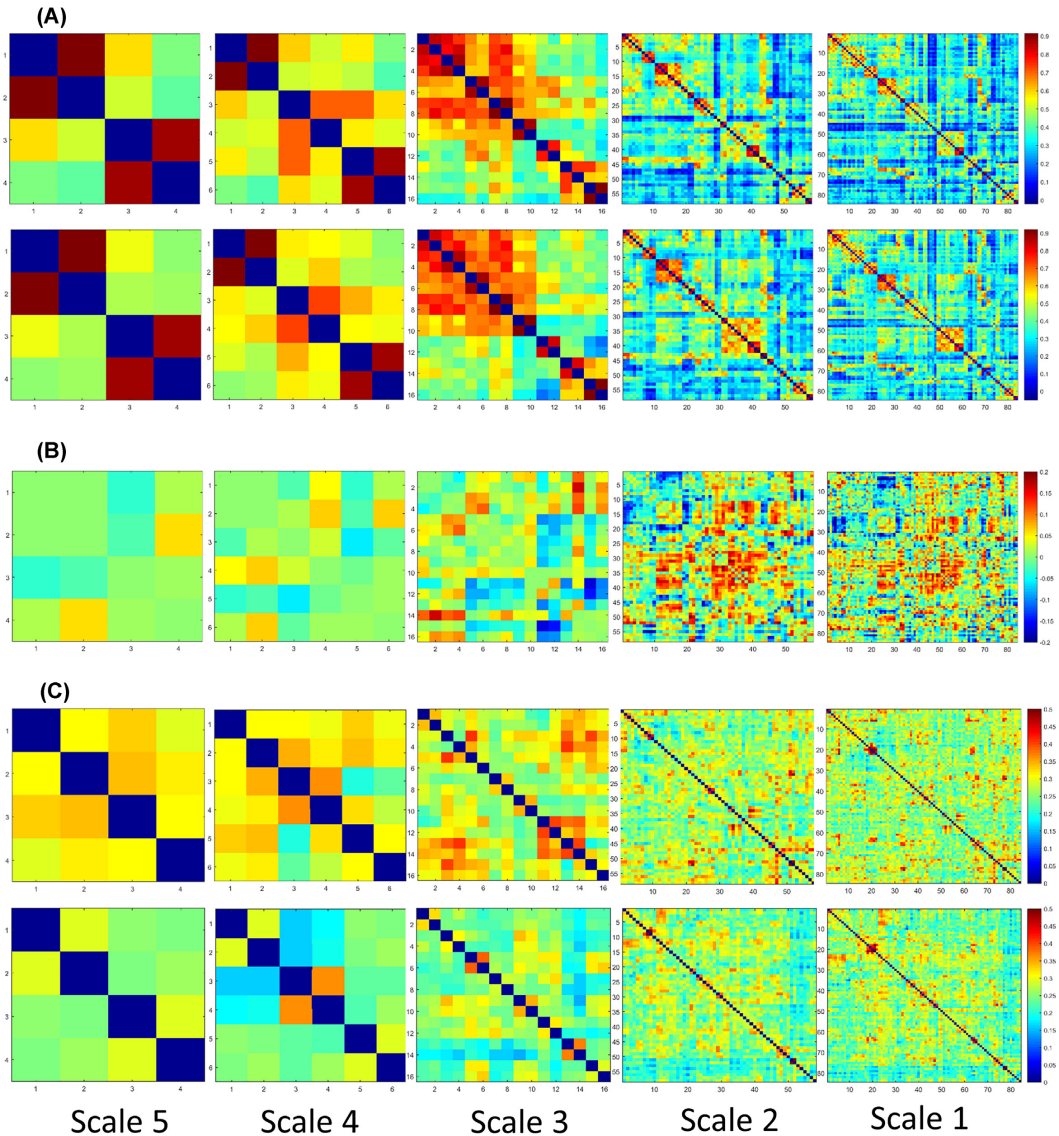
Average functional networks across the AD and NC population at five scales. (A) Average graphs of AD (row 1) and NC (row 2). (B) The difference between the average NC and average AD graphs (NC minus AD). (C) Standard deviations across the AD (row 1) and NC (row 2) populations.

Figure 2 shows a first impression of the pyramid representation of brain networks across five scales. The average graphs at each layer of the pyramid are visualized for AD and NC populations in Fig. 3A. Based on a visual inspection, the average graphs showed generally consistent patterns between the two groups. As shown in Fig. 3B, larger inter-group differences were observed in smaller-scale functional networks. The maximal inter-group difference was 0.06 (NC > AD) for Scale-5. However, this value increased to 0.23 (NC > AD) for Scale-1. Fig. 3C displays the standard deviation of the functional connectivity within each population. At Scale-5, the maximal standard deviation was 0.34 for the AD population and 0.30 for the NC population. However, at Scale-1, this value increased to 0.56 for the AD population and 0.54 for the NC population. In general, the AD population exhibited a higher standard deviation of functional connectivity than the NC population at all scales.

### Multi-scale global metrics

**Figure 4:**
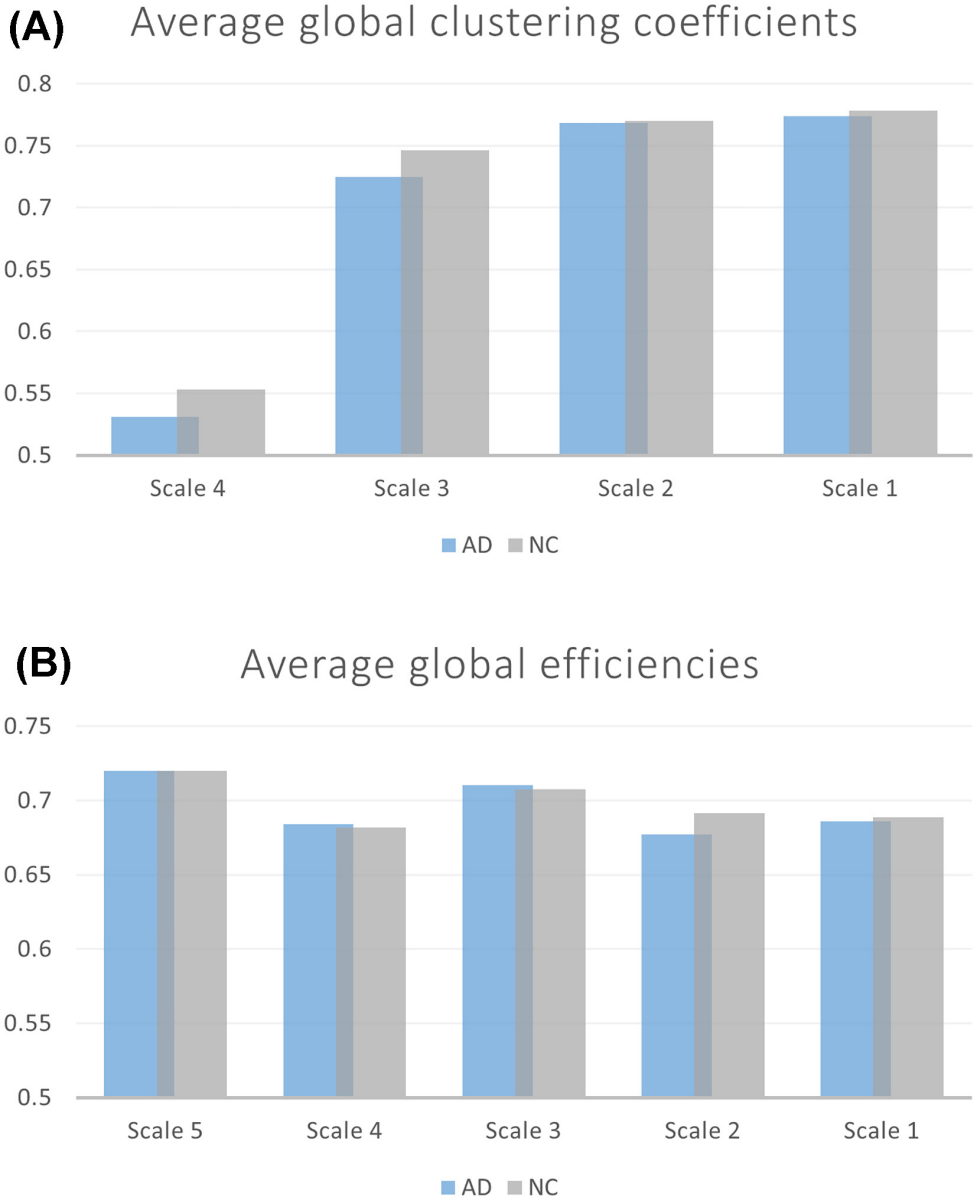
Comparison of global metrics between AD and NC at five scales. (A) The average global clustering coefficients of AD and NC. The global clustering coefficients at Scale-5 were equivalent to zeros which are thus not shown. (B) The average global efficiencies of AD and NC.

Global clustering coefficients and the global efficiencies of the networks were calculated to understand the overall capability of parallel information transfer and processing in the brain at different scales. Both metrics were averaged across the population and are displayed in Fig. 4. Higher global clustering coefficients were observed for the NC population than for the AD population at all scales. The networks of both populations showed an increasing global clustering coefficient as the scale decreased. The global clustering coefficients at the largest scale (Scale-5) were equivalent to zero in both populations, which were thus not shown for comparison in the figure. The global efficiencies of both populations were identical at Scale-5 but became randomly distributed as the scale decreased.

### Multi-scale local metrics

**Figure 5:**
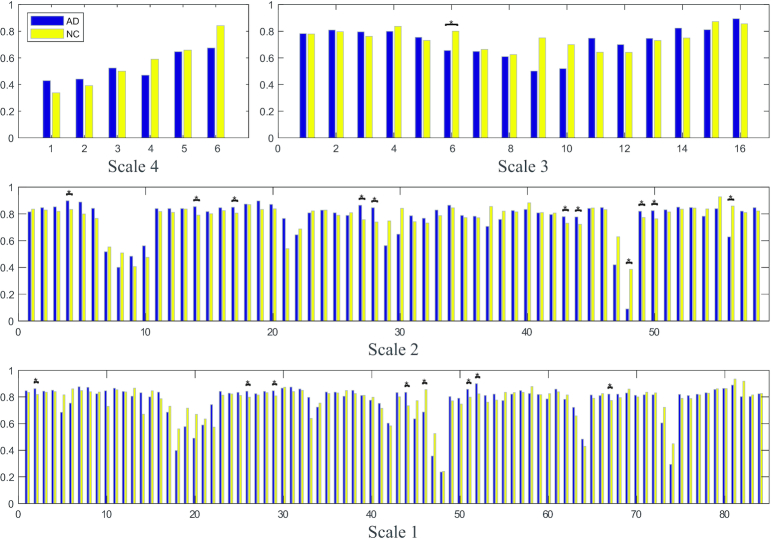
Comparison of inter-group local clustering coefficients at multiple scales. * denotes the significant differences (*P* < 0.05, uncorrected) between AD and NC. The clustering coefficients were not shown for Scale-5 since the networks yielded all zero values.

**Figure 6:**
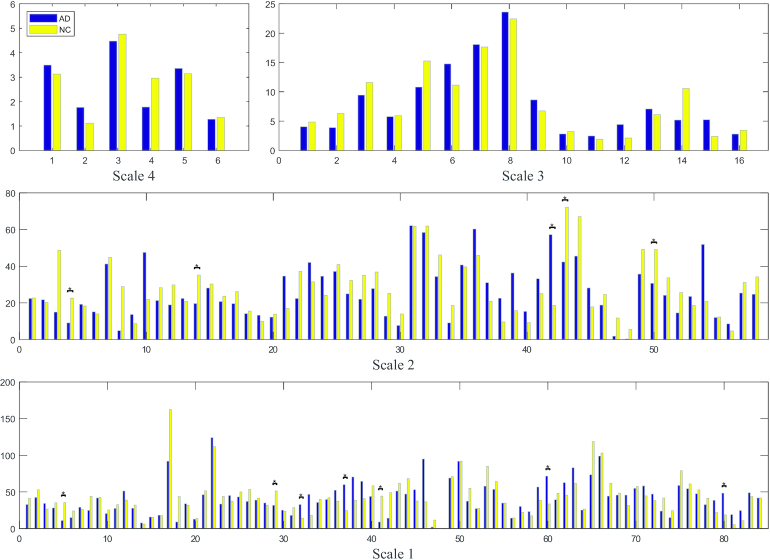
Comparison of inter-group local betweenness centralities at multiple scales. * denotes the significant differences (*P* < 0.05, uncorrected) between AD and NC. The clustering coefficients were not shown for Scale-5 since the networks yielded all zero values.

Local metrics, including the clustering coefficient, efficiency, and node betweenness centrality, were determined for each node. Using a two-tailed Student's *t*-test, we compared the inter-group differences (*P* < 0.05) in the clustering coefficients and betweenness centrality at five scales. The local metrics were not shown for Scale-5 since the networks all yielded zero values. As shown in Fig. 5, Scale-3 networks exhibited significantly higher clustering coefficients in the right temporal lobe in NC individuals than in patients with AD. Significantly higher values were also observed for the right amygdala and globus pallidus in Scale-2 networks of NC individuals. In Scale-1, significantly higher clustering coefficients were observed for NC individuals in the right parahippocampal gyrus than in patients with AD. In Fig. 6, significantly higher betweenness centrality was observed in the right middle frontal gyrus, right precentral gyrus, left cingulate gyrus, and right hippocampus at Scale-2, as well as in the left superior frontal gyrus pole, left supramarginal gyrus, and left middle temporal gyrus at Scale-1. Notably, an increasing number of inter-group differences (both positive and negative) were observed at smaller scales. The patterns of local efficiencies were very similar to clustering coefficients, and thus were not illustrated for comparison. None of these local metrics were significantly different at *P* < 0.05 after the false discovery rate (FDR) correction.

### Multi-scale metrics in the frequency domain

**Figure 7:**
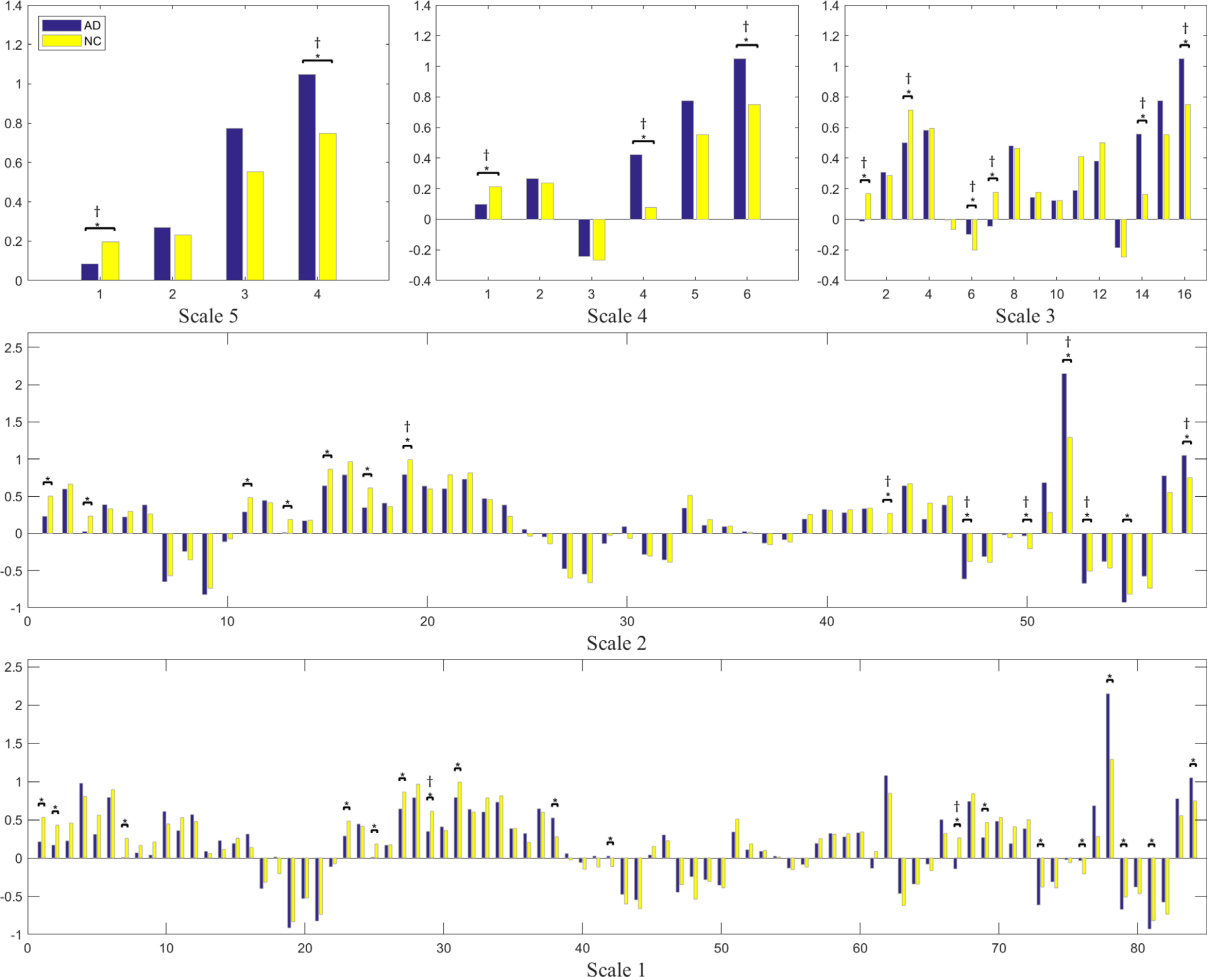
Comparison of inter-group ALFF at multiple scales. * denotes the significant differences (*P* < 0.05, uncorrected) between AD and NC. † denotes the significance (*P* < 0.05) after FDR correction.

The local metrics in the frequency domain are shown in Fig. 7 in terms of the averaged ALFF within five-scale anatomical nodes. According to the inter-group comparison, compared with AD, multiple areas showed significantly higher ALFFs in NC participants, including the left telencephalon at Scale-5; left cerebral cortex at Scale-4; left frontal lobe, left parietal lobe, and left limbic region at Scale-3; left superior/middle frontal gyrus, left pre-/post-central gyrus, left superior parietal gyrus, left supramarginal gyrus, left angular gyrus, left cingulate gyrus, left amygdala, left putamen, and left globus pallidus at Scale-2; and bilateral superior frontal gyrus, left middle frontal gyrus, left pre-/post central gyrus, left superior parietal gyrus, left supramarginal gyrus, left angular gyrus, left dorsal anterior cingulate gyrus, left post-cingulate gyrus, left amygdala, left putamen, and left globus pallidus at Scale-1. After the FDR correction, several areas lost their significance at Scale-2, including the left superior/middle frontal gyrus, left pre-/postcentral gyrus, left superior parietal gyrus, left supramarginal gyrus, and left globus pallidus. Two areas in Scale-1 survived after the correction: the left supramarginal gyrus, and left dorsal anterior cingulate gyrus.

### Validation of multi-scale metrics

The abilities of the metrics to accurately differentiate AD from NC are summarized in Table 3, including specificity, sensitivity, and accuracy rate. Among the five scales, the maximal accuracies were identified at Scale-2 for betweenness centrality, Scale-3 for the clustering coefficient, Scale-2 for efficiency, and Scale-5 for ALFF. The combined features of all scales yielded the highest classification accuracies across all metrics.The feature that maximizes the discrimination was identified as the combined ALFF, which successfully assigned 81.6% of the participants to their correct categories.

**Table 3:** Comparison of classification accuracies with respect to different local metrics at multiple scales.

	Betweenness centrality			Clustering coefficient		
	Specificity (%)	Sensitivity (%)	Accuracy (%)	Specificity (%)	Sensitivity (%)	Accuracy (%)
Scale-5	–	–	–	–	–	–
Scale-4	47.74	40.00	40.48	50.00	48.48	49.47
Scale-3	48.00	46.88	47.88	70.00	70.37	70.11
Scale-2	66.67	60.61	63.23	66.67	66.67	67.20
Scale-1	59.38	60.00	58.99	67.74	69.23	68.25
Combined	71.43	68.97	70.11	73.08	67.74	70.63

	Efficiency			ALFF		
	Specificity (%)	Sensitivity (%)	Accuracy (%)	Specificity (%)	Sensitivity (%)	Accuracy (%)
Scale-5	–	–	–	80.77	74.19	77.25
Scale-4	43.33	40.74	42.06	70.37	66.67	67.99
Scale-3	66.67	63.33	64.55	76.92	70.97	73.28
Scale-2	67.86	65.52	66.67	70.00	70.37	70.11
Scale-1	66.67	63.33	65.34	75.86	75.00	75.40
Combined	73.08	67.74	70.63	80.42	82.77	81.60

### Result of the Autism experiment

**Figure 8:**
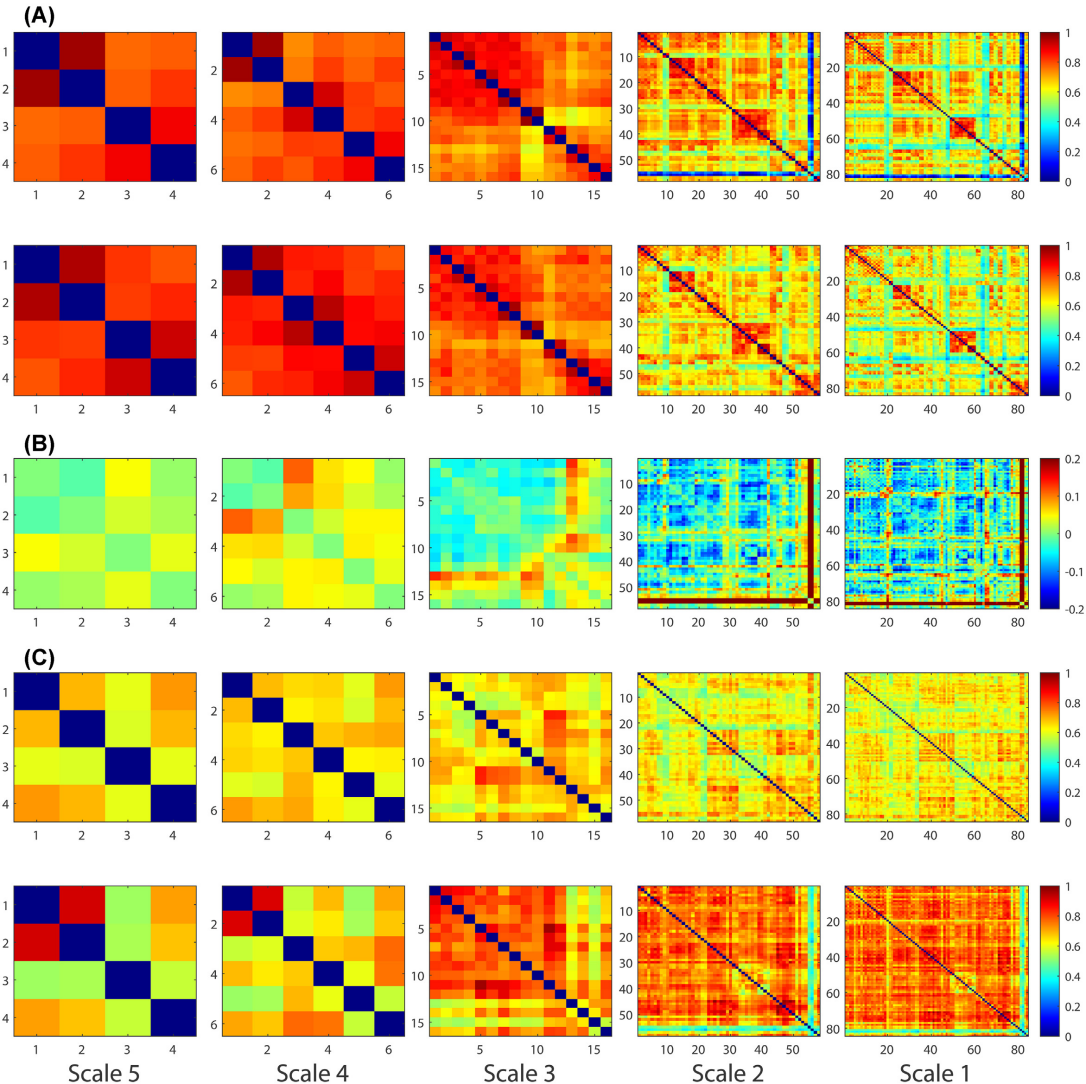
Average functional networks across the Autism and Control population at five scales. (A) Average graphs of Autism (row 1) and Control (row 2). (B) The difference between the average Control and average Autism graphs (Control minus Autism). (C) Standard deviations across the Autism (row 1) and Control (row 2) populations.

To demonstrate the reproducibility and operability of our experiments, we selected 30 people with autism and 29 normal individuals for validation. The average graphs at each layer of the pyramid are visualized for Autism and Control populations in Fig. 8A. Based on a visual inspection, the average graphs showed generally consistent patterns between the two groups. As shown in Fig 8B, lager inter-group differences were observed in smaller-scale functional networks. Figure 8C displays the standard deviation of the functional connectivity within each population. At Scale-5, the maximal standard was 0.73 for the Autism population and 0.92 for the Control population. However, at Scale-1, this value increased to 0.83 for the Autism population and 0.98 for the Control population.

**Figure 9:**
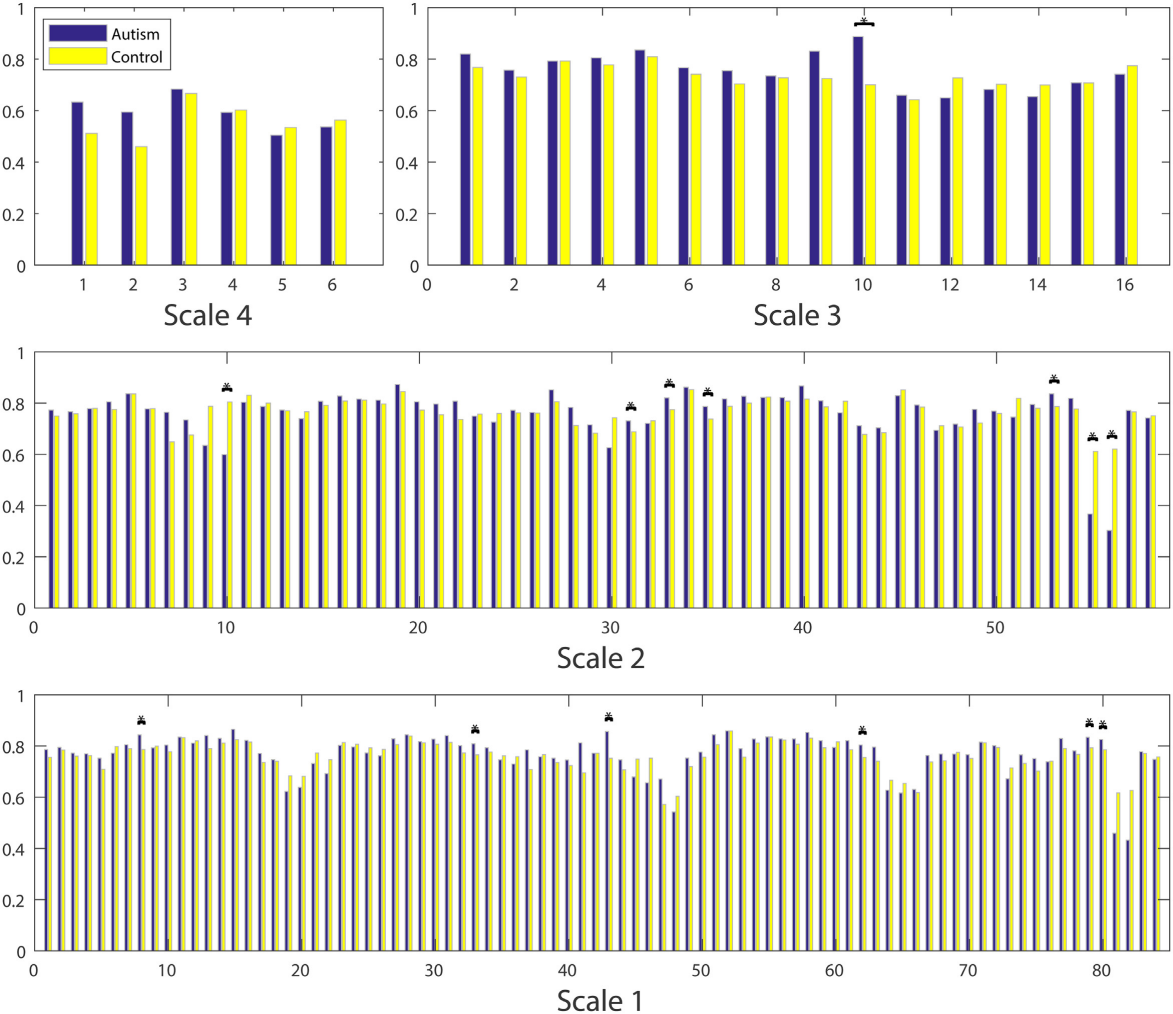
Comparison of inter-group local clustering coefficients at multiple scales. * denotes the significant differences (*P* < 0.05, uncorrected) between the Autism and Control groups. The clustering coefficients were not shown for Scale-5 since the networks yielded all zero values.

**Figure 10:**
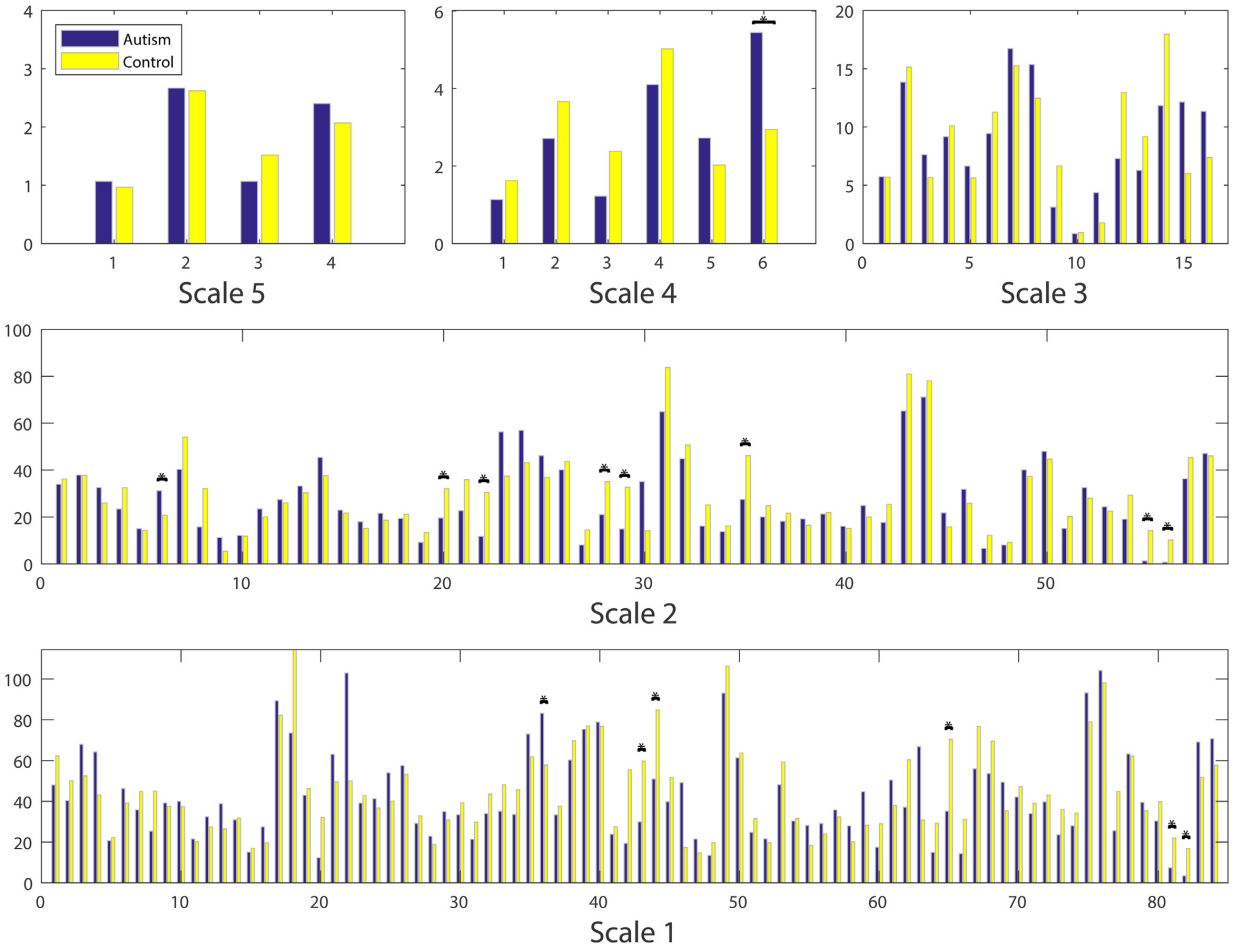
Comparison of inter-group local betweenness centralities at multiple scales. * denotes the significant differences (*P* < 0.05, uncorrected) between the Autism and Control groups.

Using a two-tailed Student's *t*-test, we compared the intergroup differences (*P* < 0.05) in the clustering coefficients and betweenness centrality at five scales. The local metrics were not shown for Scale-5 since the network yielded zero values in clustering coefficients. From Fig. 9, it can be found that the clustering coefficients of the Control group in the right gyrus rectus, bilateral globus pallidus are significantly higher than those of the Autism group at Scale-2. However, in Fig. 10, significantly higher betweenness centrality was observed in the right angular gyrus, right pre-cuneus, right inferior temporal gyrus, left limbic, left middle occipital gyrus, and bilateral globus pallidus at Scale-2, as well as in the bilateral inferior temporal gyrus, left subgenual anterior cingulate gyrus, and bilateral globus pallidus at Scale-1.

## Discussion

In this paper, we present a multi-scale representation of a human functional network by measuring the interactions between pair-wise nodes at five scales. At the largest scale (Scale-5), gray matter is parcellated into the telencephalon and diencephalon. At Scale-4, the telencephalon is then divided into cerebral cortices and cerebral nuclei. The cerebral cortices are subsequently parcellated into brain lobes at Scale-3 and further parcellated into gyrus-size ROI at Scale-2. At nine, the smallest scale (Scale-1), gyri are subdivided into smaller structures, where, for example, the inferior frontal gyrus (IFG) is parcellated to the IFG operculari, IFG orbitalis, and IFG triangularis. Smaller-sized nodes have been reported to help capture localized abnormalities. This observation was confirmed by the present study, as the smaller-scale graph showed noticeable inter-group differences that implied a loss of connectivity of susceptible areas in patients with AD. In contrast, this difference was small in the large-scale graphs. An explanation for this finding is that the average time course of a large-size anatomical structure, e.g. the cerebral cortex at Scale-4, might have insufficient sensitivity to represent the abnormal BOLD fluctuations in patients with AD. On the other hand, the small-scale graphs showed higher intrapopulation standard deviations of functional connectivities in both populations, probably due to the lower SNR and higher anatomical variability or registration error attributed to the smaller size of ROI. Moreover, the standard deviation of functional connectivities in the AD population was generally higher than in the NC population at all scales. This finding might be explained by two mixed factors: (i) patients with AD display higher anatomical variabilities in various cortical structures than NC individuals (Thompson *et al*., 1998), and (ii) the topology of AD networks has been interpreted as randomized networks due to the loss of small-worldness (Sanz-Arigita *et al*., 2010).

Based on our data, the dysfunction of local nodes and rearrangement of edges of AD networks result in reduced global clustering coefficients in patients with AD compared with NC individuals. We also observed a trend toward increased global clustering coefficients as the scale decreases. This finding is consistent with the modular structure of the brain network. In the small-scale network, nodes with denser connectivities are grouped to a module (Bullmore & Sporns, 2009). The intra-module nodes are highly connected, resulting in an increase in the global clustering coefficient, which relies on the local connectivities over the brain. However, in the large-scale networks, the group of nodes with a high density of connectivities might be included within a single node. In this case, the global clustering coefficients mainly rely on the inter-module connectivities, which are relatively weak in the network. In addition, we did not observe a trend toward a monotonic change in global efficiencies, probably due to the randomized global connectivity in both elderly and AD populations (Ohnishi *et al*., 2001).

The functional network of patients with AD might be well characterized by a feature that is highly sensitive to disease-specific dysfunction and displays the fewest redundancies. In this study, we are approaching the answer to an important question: what scale should be chosen for the optimal representation of the network? Based on the results from the present study, for local clustering coefficients, the nodes with largest inter-group differences were the right temporal lobe at Scale-3, right amygdala at Scale-2, and right parahippocampal gyrus at Scale-1. For local efficiency, the nodes with largest inter-group differences were the left cingulate gyrus at Scale-2 and middle temporal gyrus at Scale-1. These areas are consistent with previous reports of widespread patterns of gray matter degenaration or dysfunction in temporal-limbic regions and the cingulate gyrus (Christian *et al*., 2007; Ohnishi *et al*., 2001; Salat *et al*., 2011). Many metrics were reduced in the NC group compared with the AD group, mainly in smaller scale networks. Similar findings were reported in a previous study (Kim *et al*., 2015), which attributes the increased values of local metrics in patients with AD to the non-monotonic reorganization of brain networks. Inter-group differences in local features based on frequency were identified at all scales, even after the FDR correction. For different metrics, the classification rate peaked at different scales. For example, the maximal classification rate for betweenness centrality was observed at Scale-1, whereas the maximum classification rate for ALFF was observed at Scale-5. Therefore, we should be cautious when presenting the results because the comparisons involving multiple metrics are not convincing, regardless of the scale. These findings highlight the value of combining the multi-scale metrics to reconstruct a pyramid model and obtain a comprehensive representation of the network. The combine features obtained in the present study produced a higher accuracy than any single scale-based feature. The SVM enables the incorporation of many predictors into one predictive model and it automatically selects the relevant predictors. Therefore, the combined features encode the most sensitive features at every scale, where large-scale features eliminate noises, redundant information, and structural variability while small-scale features capture the localized abnormalities. This strategy has a better trade-off between feature sensitivity and anatomical variability.

In the Autism experiment, the same segmentation template from the previous experiment was used. The study found that smaller sized nodes can help capture localized abnormalities, and the smaller-scale graph showed noticeable intergroup difference, which means that a loss of connectivity of susceptible areas in patients with autism. In contrast, this difference was small in the large-scale graphs. Moreover, the standard deviation of functional connectivities in the Autism population was generally lower than in the Control population at all scales. We also observed a trend toward increased global clustering coefficients as the scale decreased.

This study has some limitations. The brain parcellation criteria are not function-specific criteria and thus might not reflect individual functional variability. For example, in the most frequently used function-specific atlas, namely the Brodmann atlas, the auditory cortex (area 41 and 42) lies in the posterior half of the superior temporal gyrus, which is defined at Scale-1 in the current study. In functional studies, the Brodmann atlas is considered more appropriate. Nevertheless, it does not offer a recombination or decomposition strategy for anatomical structures in investigations analyzing multiple scales. Moreover, a consensus regarding the gold 11 standard to divide the brain into the greatest number of functionally relevant units is still lacking. On the other hand, like previous studies (Prescott *et al*., 2014; Sanz-Arigita *et al*., 2010), the inter-group difference of the network metrics did not pass the correction for multiple comparison. However, the results still showed a trend that the dysfunctions were located at different anatomical structures at different scales. Our purpose is not to identify reliable markers according to different scales, but to demonstrate that a distinct feature identified at a scale can be used to complement the features extracted from other scales.

## Conclusion

In the present study, we present a pyramid representation of functional networks that is composed of five individual networks reconstructed at different scales. Features were extracted from the multi-scale networks, and then their discriminative power for recognizing AD was evaluated as a demonstration. Distinct features reflected the highest sensitivity of distinguishing AD at different scales. Moreover, the combined features produced higher accuracy than any single scale-based feature. These findings highlight the potential use of multi-scale features as markers of the disrupted topological organization in AD networks. We believe that the pyramid representation encodes the most sensitive feature at every scale and represents a better trade-off between feature sensitivity and anatomical variability. The presented approach thus expands our understanding of functional networks to a flexible format that could represent the underlying functional mechanism in specific population on a multi-scale basis.

## References

[bib1] Adelson EH, Anderson CH, Bergen JR et al. (1984) Pyramid methods in image processing. RCA Eng. 29:33–41.

[bib2] Barrat A, Barthé Lemy M, Pastor-Satorras R et al. (2004) The architecture of complex weighted networks. Proc Natl Acad Sci USA. 101:3747–52. 15007165 10.1073/pnas.0400087101 PMC374315

[bib3] Biswal B, Yetkin FZ, Haughton VM et al. (1995) Functional connectivity in the motor cortex of resting human brain using echo-planar MRI. MRM. 34:537–41. 8524021 10.1002/mrm.1910340409

[bib4] Bullmore E, Sporns O (2009) Complex brain networks: graph theoretical analysis of structural and functional systems. Nat Rev Neurosci. 10:186–98. 19190637 10.1038/nrn2575

[bib5] Chang CC, Lin CJ (2011) LIBSVM: a library for support vector machines. ACM Transactions on Intelligent Systems and Technology. 2:1–27.

[bib6] Christian S, Valentino R, Mark M et al. (2007) Selective changes of resting-state networks in individuals at risk for Alzheimer's disease. Proc Natl Acad Sci USA. 104:18760–5. 18003904 10.1073/pnas.0708803104 PMC2141850

[bib7] Dai Z, Yan C, Li K et al. (2015) Identifying and mapping connectivity patterns of brain network hubs in Alzheimer's disease. Cereb Cortex. 25:3723–42. 25331602 10.1093/cercor/bhu246

[bib8] Delbeuck X, van der Linden M, Collette F (2003) Alzheimer's disease as a disconnection syndrome?. Neuropsychol Rev. 13:79–92. 12887040 10.1023/a:1023832305702

[bib9] Djamanakova A, Tang X, Li X et al. (2014) Tools for multiple granularity analysis of brain MRI data for individualized image analysis. Neuroimage. 101:168–76. 24981408 10.1016/j.neuroimage.2014.06.046 PMC4165692

[bib10] Evans AC, Collins L, Milner B et al. (1991) An MRI-based stereotaxic atlas from 250 young normal subjects. Soc Neurosci Abstr. 18:408–92.

[bib11] Fair DA, Cohen AL, Dosenbach F et al. (2008) The maturing architecture of the brain's default network. Proc Natl Acad Sci USA. 105:4028–32. 18322013 10.1073/pnas.0800376105 PMC2268790

[bib12] Friston KJ, Williams S, Howard R et al. (1996) Movement-related effects in fMRI time-series. MRM. 35:346–55. 8699946 10.1002/mrm.1910350312

[bib13] He Y, Chen Z, Evans A (2008) Structural insights into aberrant topological patterns of large-scale cortical networks in Alzheimer's disease. J Neurosci. 28:4756–66. 18448652 10.1523/JNEUROSCI.0141-08.2008 PMC6670444

[bib14] Holland PW, Leinhardt S (2015) Transitivity in structural models of small groups. Comp Gr Stud. 2:107–24.

[bib15] Kim HK, Yoo K, Na DL et al. (2015) Non-monotonic reorganization of brain networks with Alzheimer's disease progression. Front Aging Neurosci. 7:1–10. 26106325 10.3389/fnagi.2015.00111 PMC4460428

[bib16] Latora V, Marchiori M (2001) Efficient behavior of small-world networks. Phys Rev Lett. 87:198701-1-198701-4. 10.1103/PhysRevLett.87.198701 11690461

[bib17] Lord C, Risi S, Lambrecht L et al. (2000) The Autism Diagnostic Observation Schedule—Generic: a standard measure of social and communication deficits associated with the spectrum of autism. J Autism Dev Disord. 30:205–23. 11055457

[bib18] McKhann G, Drachman D, Folstein M et al. (1984) Clinical diagnosis of Alzheimer's disease: report of the NINCDS-ADRDA Work Group under the auspices of Department of Health and Human Services Task Force on Alzheimer's Disease. Neurology. 34:939–44. 6610841 10.1212/wnl.34.7.939

[bib19] Ohnishi T, Matsuda H, Tabira T et al. (2001) Changes in brain morphology in Alzheimer disease and normal aging: is Alzheimer disease an exaggerated aging process?. AJNR Am J Neuroradiol. 22:1680–5. 11673161 PMC7974447

[bib20] Prescott JW, Guidon A, Doraiswamy PM et al. (2014) The Alzheimer structural connectome: changes in cortical network topology with increased amyloid plaque burden. Radiology. 273:175–84. 24865310 10.1148/radiol.14132593 PMC4263657

[bib21] Rubinov M, Sporns O (2010) Complex network measures of brain connectivity: uses and interpretations. Neuroimage. 52:1059–69. 19819337 10.1016/j.neuroimage.2009.10.003

[bib22] Salat DH, Chen JJ, van der Kouwe AJ et al. (2011) Hippocampal degeneration is associated with temporal and limbic gray matter/white matter tissue contrast in Alzheimer's disease. Neuroimage. 54:1795–802. 20965261 10.1016/j.neuroimage.2010.10.034 PMC3021138

[bib23] Sanz-Arigita EJ, Schoonheim MM, Damoiseaux JS et al. (2010) Loss of “small-world” networks in Alzheimer's disease: graph analysis of fMRI resting-state functional connectivity. PLoS ONE. 5:1–14. 10.1371/journal.pone.0013788 PMC2967467 21072180

[bib24] Song XW, Dong ZY, Long XY et al. (2011) REST: a toolkit for resting-state functional magnetic resonance imaging data processing. PLoS ONE. 6:1–12. 10.1371/journal.pone.0025031 PMC3176805 21949842

[bib25] Supekar K, Menon V, Rubin D et al. (2008) Network analysis of intrinsic functional brain connectivity in Alzheimer's disease. PLoS Comput Biol. 4:1–11. 10.1371/journal.pcbi.1000100 PMC2435273 18584043

[bib26] Thompson PM, Moussai J, Zohoori S et al. (1998) Cortical variability and asymmetry in normal aging and Alzheimer's disease. Cereb Cortex. 8:492–509. 9758213 10.1093/cercor/8.6.492

[bib27] Tzourio-Mazoyer N, Landeau B, Papathanassiou D et al. (2002) Automated anatomical labeling of activations in SPM using a macroscopic anatomical parcellation of the MNI MRI single-subject brain. Neuroimage. 15:273–89. 11771995 10.1006/nimg.2001.0978

[bib28] Valencia M, Pastor MA, Fernández-Seara MA et al. (2009) Complex modular structure of large-scale brain networks. Chaos An Interdiscip J Nonlinear Sci. 19:23119. 10.1063/1.3129783 19566254

[bib29] Wang J, Wang L, Zang Y et al. (2009) Parcellation-dependent small-world brain functional networks: a resting-state fMRI study. Hum Brain Mapp. 30:1511–23. 18649353 10.1002/hbm.20623 PMC6870680

[bib30] Wang J, Zuo X, He Y (2010) Graph-based network analysis of resting-state functional MRI. Front Syst Neurosci. 4:1–14. 20589099 10.3389/fnsys.2010.00016 PMC2893007

[bib31] Zang YF, Yong H, Chao-Zhe Z et al. (2007) Altered baseline brain activity in children with ADHD revealed by resting-state functional MRI. Brain Dev. 29:83–91. 16919409 10.1016/j.braindev.2006.07.002

